# Can the Initial Parameters of Functional Scales Predict Recovery in Patients with Complete Spinal Cord Injury? A Retrospective Cohort Study

**DOI:** 10.3390/diagnostics14020129

**Published:** 2024-01-06

**Authors:** Krzysztof Wasiak, Justyna Frasuńska, Beata Tarnacka

**Affiliations:** 1 Department of Rehabilitation, Mazovian Rehabilitation Center STOCER, 05-520 Konstancin-Jeziorna, Poland; kwasiak@stocer.pl; 2 Department of Rehabilitation, Medical University of Warsaw, 02-637 Warsaw, Poland; beata.tarnacka@wum.edu.pl; 3 Department of Rehabilitation, National Institute of Geriatrics, Rheumatology and Rehabilitation, 02-637 Warsaw, Poland

**Keywords:** tetraplegia, paraplegia, early spinal cord injury, functional scales, motor score of AIS, Barthel Index, SCIM, predictors for improvement, neurorehabilitation

## Abstract

Regaining greater independence in performing daily activities constitutes a priority for people with tetraplegia following spinal cord injury (SCI). The highest expectations are connected with the improvement of hand function. Therefore, it is so important for the clinician to identify reliable and commonly applicable prognostic factors for functional improvement. The aim of this study was to conduct an analysis to assess the impact of initial functional factors on the clinical improvement in patients during early neurological rehabilitation (ENR). This study assessed 38 patients with complete SCI aged 17–78 who underwent ENR in 2012–2022. The analysis included the motor score from the AIS (MS), the Barthel Index (BI) and the SCIM scale values at the beginning of the ENR program and after its completion. During ENR, patients achieved a statistically significant improvement in MS, BI and SCIM. The initial MS and the level of neurological injury constituted the predictors of functional improvement during ENR. Significant statistical relationships were observed primarily in the correlations between the initial MS and BI, and the increase in the analyzed functional scales of SCI patients. Higher initial MS may increase the chances of a greater and faster functional improvement during ENR.

## 1. Introduction

Spinal cord injury (SCI) results in impaired or absent motor and sensory function in segments below the level of injury (plegia). Damage to the spinal cord may result from traumatic (abrupt traumatic blow to the spine) and non-traumatic processes (slow internal damage to the spinal cord region). SCIs occur more frequently in men at a mean age of approximately 39 years. The most common causes of traumatic SCIs are road traffic accidents and tripping, while the non-traumatic causes of SCIs include degenerative diseases, tumors and infections. Depending on the level of injury, i.e., cervical or thoraco-lumbar, the extent of the motor and sensory deficits may be defined as tetraplegia or paraplegia. Injuries may be divided into incomplete and complete, depending on the presence or absence of any motor/sensory function. Incomplete cervical SCIs are the most common and complete cervical SCIs are the rarest [1]. These rarest SCIs are at the same time the most severe and result in serious consequences for the patient. Tetraplegia is associated with numerous consequences in various aspects of human life (medical, social, family and professional); it disrupts the current functioning of patients and introduces serious changes. SCIs are a cause of disability, especially among younger people, and have a large impact on the number of years lived with disability [1,2].

The natural course and the prognosis for SCIs depend on the level of SCI, and the extent and severity of the injury [3,4,5]. The “spinal shock” period should also be mentioned. This is defined as a transient loss of reflexes below the level of spinal cord injury. The duration of “spinal shock” depends on the recovery of reflexes and lasts from a few hours to several months. Clinical improvement may be predicted in patients with incomplete SCIs associated with the sensation preserved in the S4–S5 sacral segments [6], while in those with complete SCIs (AIS-A), functional improvement is less commonly observed. According to the literature, complete to incomplete SCI conversion is estimated to range from 4 to 25% [7]. It occurs mainly within the first 6–9 months after the injury (with the fastest rate of improvement in the first 3 months after the SCI) and occurs more often in cases of tetraplegia than paraplegia [3,7]. However, it should be remembered that the experience of the clinician (avoiding misdiagnoses) and the time from the SCI to the initial SCI patient examination are important in the functional assessment. According to the literature, the conversion was significantly more likely in patients who were examined within an early timeframe from SCI (four hours) compared to individuals examined afterwards [3].

Patients’ expectations are high in regard to recovery, walking and good functioning in their socio-professional lives. Restoring hand function (grasping small objects) and regaining greater independence in performing everyday activities is a priority for people with tetraplegia [8]. All reports concerning a favorable neurological prognosis or the recovery of distal motor function are desirable for patients in such catastrophic life situations and bring hope of a good prognosis. Therefore, from the clinical point of view, it is very important to identify reliable factors of clinical improvement after SCI. The ability to predict recovery after SCI is an important role of the medical rehabilitation physician, who is provided with the tools to navigate difficult conversations with patients about their chances of clinical improvement or regaining independence. Moreover, it may help to improve the strategy of medical treatment in order to achieve the best possible therapeutic effect; it facilitates setting rehabilitation goals and planning rehabilitation programs. It may also help to manage health care finances in terms of the needs of people with SCIs.

According to the literature, a number of clinical, imaging and therapeutic factors may have an impact on clinical improvement in patients with SCIs [9,10,11,12,13,14]. No publication was found in which the effects of various functional factors of the patient (scales: American Spinal Cord Injury Association (ASIA) Impairment Scale (AIS) [15], the Barthel Index (BI) [16] and Spinal Cord Independence Measure, version III (SCIM) [17]) were analyzed in detail in reference to the possibilities of clinical improvement in patients with complete SCIs (AIS-A) in the cervical segment of the spine. All these scales describe the functional status of patients after SCI and are simple tools for assessing patients’ SCIs. However, they require skill and proficiency on the part of the investigator. The AIS and SCIM scales are specific to SCIs and the BI scale is less specific to SCIs. Complete SCIs in the cervical segment of the spine (tetraplegia) occur the least frequently and such SCI patients are the most severely affected group. They face the greatest functional limitations, more severe disabilities and greater dependence on others.

The aim of this study was to analyze the impact of functional factors on the clinical improvement in patients with AIS-A during early neurological rehabilitation (ENR), with particular emphasis on the motor score of AIS (MS).

## 2. Material and Methods

### 2.1. Study Setting and Ethics Statement

This retrospective cohort study was conducted at the Prof. Marian Weiss Mazovian Rehabilitation Center STOCER in Konstancin Jeziorna, Poland. It is a center for the multidisciplinary treatment of patients after SCI covering the central region of Poland (Mazovia and the neighboring regions of the province).

The cohort study design was approved by the Ethics Committee of the Regional Medical Chamber in Warsaw (Poland) (No. KB/1462/23, OKW/8416/2023).

### 2.2. Study Population

We retrospectively collected the data of patients with SCI. All patients included in this study experienced SCI in the cervical segment of the spine and underwent early rehabilitation in the years 2012–2023 in the Department of Neurological Rehabilitation of the Mazovian Rehabilitation Center STOCER, Konstancin Jeziorna, Poland.

The inclusion criteria comprised:

- Patients with complete quadriplegia (AIS-A);
- Patients after surgery due to a SCI (decompression and stabilization);
- Patients admitted directly from the acute phase center (spinal surgery unit or intensive care unit) to the ward providing ENR.

The exclusion criteria comprised:

- Patients who suffered peripheral nerve damage in the upper limbs along with SCI;
- Patients who suffered other severe injuries of the upper limbs or their amputation along with SCI;
- Patients who had suffered a central nervous system injury in the past resulting in paresis (partial loss of motor function) or plegia (complete loss of motor function);
- Patients who had already had paresis or plegia of limbs in the course of chronic diseases prior to their SCI.

### 2.3. Patient Selection and Data Collection

**Figure 1:**
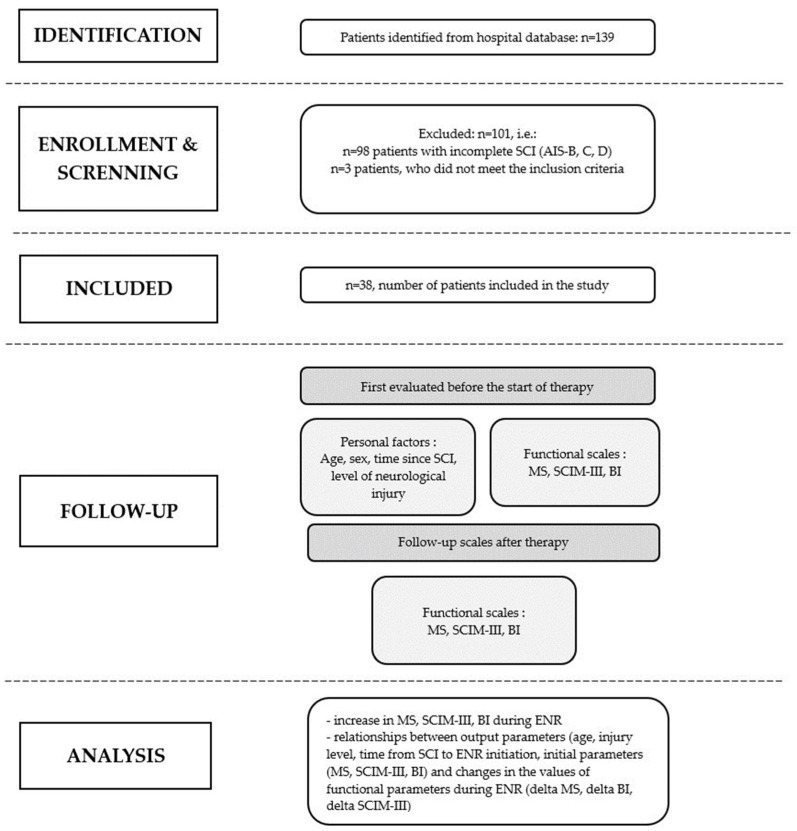
A flowchart of patient recruitment and study conduct.

The flow chart of the retrospective study cohort of patients with early SCI who underwent ENR is presented in Figure 1. The analysis included the course of hospitalization of patients meeting the criteria described previously. It concerned the medical records of patients.

### 2.4. Early Neurological Rehabilitation

All patients were transferred to the rehabilitation unit directly from the acute center (i.e., a neurosurgical ward, intensive care unit, etc.) in the shortest possible time. The stays of patients with complete cervical SCIs in the acute center were generally longer than patients with other grades of SCI due to the severity of the injury and the presence of secondary health problems. The criteria for admission to the rehabilitation unit were the good, stabilized clinical condition of the patient, verified by the doctor of physical and rehabilitation medicine. The patient’s prolonged stay in the acute phase unit was limited by the patient’s unstable medical condition, their cardiopulmonary capacity or possible early SCI complications requiring continuous monitoring and direct patient supervision.

Patients who were admitted to the rehabilitation unit had a standard program of rehabilitation provided to patients with SCIs in the early period after an injury (ENR). The stay in the ENR department usually lasted 16 weeks. The upper limit of the stay of SCI patients in the department was related to the limits of reimbursement of state healthcare for SCI patients under the National Health Fund in Poland. The limit of the patient’s stay in the unit was determined by the doctor of physical and rehabilitation medicine on the basis of the patient’s clinical condition, and their ability, mobility, functional capacity and readiness to undertake further rehabilitation steps, including environmental and occupational readaptation.

The majority of the patients in our study had long-lasting ENR. For one patient, the stay was interrupted for medical reasons and the patient was referred to an acute phase center, and he did not return to our unit. Conversely, for another patient, the stay was prolonged due to the patient’s severe social situation and difficult housing conditions.

While in the neurological rehabilitation ward, patients have a physiotherapy program (as below) for six days a week and are subject to 24 h treatment (inpatients), during which the other medical interventions listed below are provided. As part of the rehabilitation program, each patient underwent physiotherapy: individual training based on physiotherapeutic methods, active and passive exercises, passive ambulation, adaptation to a manual wheelchair, learning to transfer from a wheelchair to a bed and vice versa, and full-body training.

In addition, the inpatient treatment program included:

- Urological management: regulation of urination, intermittent catheterization/cystostomy, learning appropriate behaviors, pharmacotherapy and prevention/treatment of urinary tract infections;
- Treatment of concomitant spasticity: physiotherapy, pharmacotherapy and learning appropriate behaviors;
- Prevention/treatment of cardiorespiratory complications: respiratory infections, orthostatic hypotonia, autonomic dysreflexia and venous thromboembolism (bronchial toilet, physiotherapy, pharmacotherapy and learning appropriate behaviors);
- Prevention/treatment of intestinal complications, management of bowel movements: dietary treatment, physiotherapy, pharmacological management and learning appropriate behaviors;
- Anti-bedsore prophylaxis: changing body position, using pressure-relieving devices, i.e., mattress, wheelchair cushion and learning appropriate behaviors;
- Pain management: pharmacotherapy and physiotherapy;
- Others, e.g., treatment of concomitant diseases (according to patients’ needs).

### 2.5. Research Tool and Outcome Measures

The functional status of patients was assessed at the beginning and at the end of the ENR using the following scales: AIS, BI and SCIM (Figure 1). The examination was performed by physicians with at least two years of experience in the treatment of patients with SCIs, trained in conducting a neurological examination in accordance with the requirements of the international standards for the neurological classification of SCIs.

The AIS [15] is a generally accepted/standard functional scale for SCI patients. It is based on bilateral testing of the muscle strength of the upper and lower limbs (motor score from the AIS (MS)) supplied by the spinal nerves from C4 to Th1 and from L2 to S1, and bilateral testing of the sensation of touch and pain within 18 dermatomes from C2 to S4/S5. The final score of the scale is calculated on the basis of points obtained for muscle strength (from 0 to 100 points) and sensory testing (from 0 to 112 for touch sensation and from 0 to 112 for pain sensation). A total of 50 motor points from AIS apply to the upper limbs and 50 to the lower limbs. The score is calculated from a muscle strength test (range 0 to 5) of five muscles from the cervical spine level (C4 to Th1) and five from the lumbosacral spine level (L2 to S1). Based on the obtained score, patients are classified into the following groups, ranging from complete loss of neural function below the injury to completely normal: complete SCI, i.e., AIS-A group (meaning the lack of motor and sensory activity below the level of injury, including the lack of sensation in the S4/S5 segments) and incomplete SCI, with patients being assigned to different groups (AIS-B, AIS-C, or AIS-D) depending on the obtained score [15]. To determine the level of neurological injury, we specify the highest level with normal muscle strength and sensation, below which neurological defects occur.

The Barthel Index (BI) [16] is not SCI-specific, but it allows the functional assessment of a patient with a disability and their need for care/assistance from others. On the basis of the BI, a score of 0 to 20 points may be obtained following the assessment of ten parameters including: the control of anal sphincters (from 0 to 2 points), the control of bladder sphincters (from 0 to 2 points), grooming (from 0 to 1 point), using the toilet (from 0 to 2 points), eating meals (from 0 to 2 points), transfer (from 0 to 3 points), mobility (from 0 to 3 points), dressing/undressing (from 0 to 2 points), walking up the stairs (from 0 to 2 points) and personal hygiene/bathing (from 0 to 1 point) [16].

The SCIM [17] scale is a functional scale dedicated to patients with SCIs. It is a self-explanatory questionnaire used to determine the extent to which a person with a SCI can perform specific tasks on their own. The SCIM consists of seventeen elements assessing four domains: 1. Self-care, 2. Respiration and sphincter management, 3. Mobility and 4. Locomotion. The following items are assessed within each domain:

1. Self-service: feeding and bathing (upper and lower body half), dressing (upper and lower body), hygiene, grooming and caring for the appearance;
2. Respiration and sphincter management: assisted or unassisted breathing, urination and the assessment of residual urine volume, regularity/irregularity of bowel movements, assisted bowel movements and use of the toilet;
3. Mobility: changing position in bed and preventing pressure sores, independent/assisted transfer from wheelchair to bed and vice versa, and independent/assisted transfer from wheelchair to bathtub;
4. Locomotion: inability to move, assisted transfer, e.g., via wheelchair, rehabilitation devices, depending on the distance (>10 m, 10–100 m, <100 m), the possibility of walking up the stairs, and transfer from wheelchair to car and from the floor to wheelchair.

SCIM total scores range from 0 to 100. The original scale was improved several times. Currently, version III is used [17].

We analyzed factors (data from patients’ medical history) that might affect the prognosis regarding possible functional improvement during ENR.

The analysis included:

- Initial parameters: age, time elapsed from SCI to ENR initiation (corresponding to the length of the acute stay), the level of neurological injury and initial (before the start of ENR) parametric values of the MS, BI and SCIM scales
- Final parameters (after ending ENR): parametric values of the MS, BI and SCIM scales;
- Change of functional parameters: increase in MS, BI and SCIM functional parameters in the course of ENR;
- Correlations between the initial values of scales (MS, BI and SCIM), age, time since SCI and the level of neurological injury, and changes in functional parameters in the course of ENR.

### 2.6. Statistical Methods

The collected data were summarized using the mean and standard deviation for normally distributed continuous variables or the median and interquartile range (IQR) for skewed distributed continuous variables. The distribution of quantitative variables was presented by means of histograms and, in short, as medians with an interquartile range due to the ordinal character of the variables of interest (MS, BI and SCIM). A *p*-value of 0.05 was regarded as significant. Changes in the MS, BI and SCIM scales (initial vs. final ENR) were tested with the signed ranks test. The relationships and strengths between quantitative variables (MS, BI and SCIM) were assessed using correlation analysis and the Spearman’s correlation coefficient, respectively. All calculations were performed using the SAS System, version 9.4, Cary, NC, USA.

## 3. Results

### Participants

In the years 2012–2022, a total of 139 patients with SCIs at the cervical level of the spine were hospitalized in STOCER, Konstancin-Jeziorna, Poland. In this group, complete SCI was confirmed in 41 patients (AIS-A) and incomplete SCI (AIS-B, C, D) in 98 patients. Of the 41 subjects with complete SCI, 38 met the inclusion criteria. Three individuals with complete SCI were not enrolled in the cohort study: one patient had a stroke in an acute phase center, one had a cerebellar stroke that occurred in an acute phase center, and one had a SCI with a concomitant brachial plexus injury.

The study group included 38 patients with AIS-A aged 17 to 78 years. The characteristics of the group with complete SCIs are presented in Table 1.

The values of functional parameters before and after rehabilitation are presented in Table 2.

**Figure 2:**
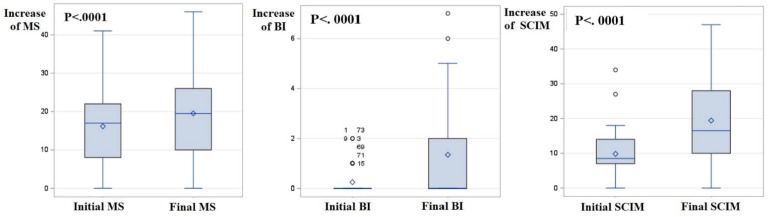
Graphical representation of changes in functional scale parameters during early neurological rehabilitation. Abbreviations: BI: Barthel Index; MS: ASIA (American Spinal Cord Injury) Scale Motor Scores; SCIM: Spinal Cord Independence Measure, version III.

The analysis based on SCI functional scales revealed a statistically significant functional improvement in the increase in the MS, BI and SCIM scores (Table 3, Figure 2). None of the enrolled patients was found to convert from AIS-A (initial assessment prior to ENR) to AIS-B, C, D or E (final assessment after ENR). Moreover, no change in the level of neurological injury was observed during ENR for any patient enrolled in this study.

Initial MS, initial BI, initial SCIM, age and level of SCI were found to be statistically significant predictors of clinical improvement during ENR, as expressed by patients achieving higher values on functional scales.

Higher initial MS values correlated with greater BI increases (r = 0.65, *p* < 0.0001) and greater SCIM increases (r = 0.36, *p* < 0.028). Higher BI initial values correlated with greater MS increases (r = 0.34, *p* < 0.039) and greater BI increases (r = 0.39, *p* < 0.017). Higher initial SCIM values correlated with greater BI increases (r = 0.38, *p* < 0.018).

Longer stays in an acute phase treatment center (time from SCI to ENR initiation) correlated negatively with BI increases (r = −0.50, *p* < 0.002). Patients staying in the acute phase treatment center for longer periods achieved smaller increases in BI than those who had shorter stays.

A lower level of SCI correlated with greater BI increases (r = 0.46, *p* < 0.004). Patients with lower levels of SCI achieved higher BI values during ENR.

The remaining initial parameter correlations showed no statistical significance with the increase in functional scale values during ENR (Table 4).

A statistical correlation analysis was also performed between the individual initial parameters (age, sex, level of injury, time from SCI to ENR initiation and duration of ENR). This analysis showed statistical significance only between the level of SCI and the length of stay of the patient in the acute phase treatment center (r = −0.38, *p* < 0.016). Patients who had a lower level of neurological injury stayed in the acute phase treatment center for a shorter time than patients with a higher level of neurological injury.

## 4. Discussion

To our knowledge, this is the first study that analyzes the prognostic factors of clinical improvement in detail based on available scales (AIS, BI, SCIM) in such a homogeneous group of patients (AIS-A) with early SCI.

In our study, MS on admission to ENR appeared to be a predictor of increased BI and SCIM in the course of ENR. High BI prior to the start of ENR proved to be a predictor of improved MS and BI during ENR. High initial SCIM values correlated with greater improvements in BI values during ENR. Also, lower levels of neurological injury prognosticated greater BI improvements.

### 4.1. Functional Parameters

The improvement in the functional parameters of patients after ENR which we observed in our study constitutes a kind of confirmation of the effectiveness of the treatment and therapeutic procedure, and confirmation of what is known and proven by previous studies, i.e., that the clinical and functional condition after a SCI improves during ENR [18]. This was also confirmed in electrophysiological tests [19]. Rehabilitation may contribute to an increased spontaneous plasticity within segmental spinal cord circuitry. Rehabilitation-driven neural plasticity was found to create the ability to guide connections to create more normal functions [20].

#### 4.1.1. Motor Score from ASIA Impairment Scale

The AIS is a standardized neurological examination for SCI patients. Based on motor, sensory and anorectal examinations of patients, injury severity or grade and level are determined, and patients are assigned to a specific grade of SCI. Complete SCI in the cervical segment of the spine (AIS-A grade) represent the most severe form of injury.

In regard to available publications, similar to our study, a study by Fisher et al. [7] included a homogeneous group of patients with AIS-A and presented comparable study results, i.e., distal motor function was not regained. The researchers noted that an improvement in the MS was not associated with the neurological level of injury (C0–C4 vs. C5 vs. C6–C7) in complete cervical SCIs. However, the study determined other analyzed parameters and did not analyze the relationship between functional scales in such detail. Moreover, the functional status was determined using other scales, i.e., the functional independence measure (FIM) scale was used in addition to the AIS. The FIM was developed as a measure of disability and includes measures of independence for self-care, sphincter control, transfer, locomotion, communication and social cognition. In addition, the methodological assumptions of the researchers were different, motor recovery in the first three levels caudal to the motor level was measured and defined as “local recover” [7] and the patients included in the study experienced SCI two years before. Therefore, it is difficult to make a clear comparison between this study and ours. Furthermore, the FIM scale is mainly applicable to the measurement of the burden of care, which does not necessarily reflect the functional recovery of the patient [21].

Similar scales (the MS and FIM) were used in another study that included patients with cervical SCIs regarding the disproportionately greater weakness in the upper limbs compared to the lower limbs and involved a sacral pinprick or voluntary motor sparing [22]. The initial MS score was also an important predictive variable in the study, along with other important predictors such as: education, comorbidities, age and spasticity. However, the methodological differences between the above-mentioned study and our study in relation to the scales used and the observation time (at least two years after SCI) make them difficult to compare.

Despite the observed methodological differences in the studies determining the predictors of clinical improvement, the initial MS is classified as a prognostic factor of functional improvement after SCI [9,10,23]. The advantage of this parameter is linked to the fact that it is a relatively simple tool for examining SCI patients, as it only requires the skill of the examiner. According to Mputu Mputu et al. [10], the MS belongs to category A (high evidence): consistent quality of evidence of a significant effect of the predictor on the outcome [10]. Our study also confirmed the importance of this parameter in the process of predicting clinical improvement. Other studies showed that SCI patients with a higher score on the initial MS were more likely to achieve better outcomes at follow-up [22,24]. However, some authors suggested that the use of the ASIA motor subscales for the upper and lower limbs could predict the results more effectively than the use of the total MS score [25]. A large retrospective analysis of 748 individuals from the European Multicenter Study about SCI revealed that the prediction of strength recovery by the initial MS was poor (R^2^ = 0.148) in patients with complete SCI [26]. Individuals classified as AIS-A or B exhibit some degree of proportional recovery of the upper limb muscles if the initial impairment is low, while the change/improvement in strength is constant or inversely proportional if the initial impairment is high (especially for distal hand muscles) [26].

#### 4.1.2. The Barthel Index

The BI is a 20-point scale allowing for the functional assessment of a patient with a disability and their need for care and assistance from others. The authors found no publication that analyzed the BI scale in relation to predictors of clinical improvement. However, the BI is not a scale dedicated to SCIs and rarely appears in publications concerning SCI patients [27,28]. Moreover, research showed the minimal importance of the BI in the assessment of functional outcomes for patients with SCIs [21]. Nevertheless, we observed some statistical relationships in the analysis of predictors of clinical improvement in relation to the BI. Thus, perhaps this parameter, despite its inaccuracy, may constitute a potential prognostic factor in the early period after a SCI.

#### 4.1.3. Spinal Cord Independence Measure

The SCIM is used to assess the ability of patients after a spinal cord injury to perform basic activities of daily living independently. It focuses on self-care, respiration and sphincter management, mobility and a patient’s locomotion.

The AIS and SCIM are multidirectional scales that assess functional status. The SCIM evaluates the patient’s ability to perform daily activities independently [29], while the AIS score evaluates the patient’s sensory and motor functions. The SCIM may be a good parameter for the verification of functional improvement, especially in the early period after a SCI [30]. However, different interpretations of this parameter in relation to the level of injury (tetraplegia vs. paraplegia) may interfere with the final conclusion of this study [31]. Van Hendel and Curt described a high correlation between the AIS-A motor score and the total SCIM score (r = 0.63, *p* < 0.001) in patients with tetraplegia, but this was significantly lower in patients with paraplegia (r = 0.26, *p* = 0.06). This may indicate some limitations in the use of this measurement tool in the functional assessment of patients with trauma below the cervical segment of the spine. Our study showed that initial MS is a predictor of SCIM score improvement in the course of ENR. We do not know how long after SCI other researchers conducted their studies. In our study, the initial SCIM did not show a statistical relationship in regard to the increase in the MS, but such a relationship was observed in relation to delta BI. Few studies took account of the SCIM scale in the analysis of predictors of clinical improvement [32]. The study by Wichmann et al. [32] revealed a negative correlation between the presence of comorbidities, clinical progression according to the AIS, MS and the time of spinal decompression, and the degree of functional independence assessed by the SCIM up to 1 year after SCI. However, it is important to note that respiration and sphincter management (components of the SCIM scale) are not domains directly related to the MS of the upper limbs. In addition, the FIM scale is more commonly used in publications to assess functional status [9,10], which makes comparisons between studies more difficult.

### 4.2. Other Parameters

In our study, age was not a significant predictor of clinical improvement in correlations with scales. Conversely, the time from SCI to ENR initiation proved to be significantly correlated with an improvement in BI results. The time since SCI was negatively correlated with the BI. The negative correlation may be explained by the originally more severe condition of patients resulting in the need for a longer stay in the neurosurgery department or ICU. The longer the time from SCI to the start of ENR, the lower the BI increases were. This result is not surprising given that our study also showed a correlation of time in the acute phase treatment center with the level of neurological injury. Patients with higher levels of SCI tended to have more difficulty returning to at a minimum necessary respiratory capacity.

Meanwhile, the literature results vary. According to a review by Mputu Mputu et al., the influence of the patient’s age on the results of functional improvement remained largely unconfirmed. However, the inconsistency or heterogeneity of studies included in the analysis was emphasized, which classified this predictor in the category of moderate evidence. In the review, the “time since SCI” predictor was placed in a similar category [10]. Conversely, the initial severity of SCI based on the AIS, MS or neurological injury level were the strongest predictors of clinical/functional improvement in SCI patients in this publication. Patients with more severe injuries on admission showed poor neurological outcomes [10]. The results partially overlap with those of our study. According to other authors, motor and functional regeneration of SCI patients decreased with age in case of complete SCI, and the severity of SCI was the most significant predictor of improvement [9]. However, both reviews included SCI patients with various AIS scores (from AIS-A to AIS-D), which differentiates them from our study, making a clear comparison impossible.

### 4.3. Conversion from Complete to Incomplete SCI

The conversion from AIS-A to incomplete SCI, described in the literature in different percentages, i.e., in the range of 4–25% [7], was not confirmed in our study. Such a result may indicate the extensive experience of our physicians in the clinical assessment of a patient with SCI. It may also be the result of the small size of our study group or the differences in the methodology of other studies describing such conversions. Undoubtedly, the analysis of the conversion of complete SCI to incomplete SCI should also take account of the fact that the determination of the initial AIS parameter prior to ENR initiation is performed in the early period following SCI, i.e., during spinal shock. This may be associated with potential measurement errors resulting from the specificity of symptoms during this SCI period or with other confounding factors.

## 5. Strengths and Limitations of This Study

### 5.1. Strengths of This Study

Previous studies described in the literature did not include an analysis of the relationship of all the above mentioned clinical improvement factors to the extent we did. The majority of studies evaluating the predictors of clinical improvement were conducted in heterogeneous groups of complete and incomplete SCI patients [9,10]. Some authors also provided reports which included non-traumatic SCIs [33]. Despite similar clinical effects that are observed after ENR for traumatic and non-traumatic SCIs [34], subtle differences may occur between them that make their course, prognosis and final ENR outcomes different. In our study, all the patients experienced traumatic SCIs. The homogeneous study group (complete SCI from the cervical injury of spinal cord) is, undoubtedly, a strength of our study.

In addition, all patients were examined by an experienced team of doctors (two years of experience in the treatment of patients with SCIs). This was unquestionably helpful in eliminating possible errors due to an incorrect classification into AIS groups. No conversion from complete SCI to incomplete SCI was observed in our group, which may indicate the extensive experience of our physicians in the clinical assessment of patients with SCIs.

Furthermore, the functional scales used in this study are reliable, well known and do not require special instrumentation or complex calculations. They are well verified and widely available in centers for SCI treatment around the world. In addition, the analysis of not one, but three functional scales gives a more complete picture of the possibilities for functional improvement.

In addition, this study used adequate statistical analysis methods, which improved the quality of this study.

### 5.2. Limitations of This Study

The authors declare that this study was conducted in one center and, undoubtedly, a deeper analysis of the described problem would require multicenter or international research.

In addition, a small group of patients may constitute a limitation of this study. The inclusion of more patients would require the analysis across a longer time span or conducting multicenter research.

Other factors, such as the level of spasticity, pain, type of surgical treatment and the time of its implementation after SCI, the cause of SCI, education, and social and professional status, were also not subject to analysis [10]. A broader analysis of these issues might present our study in a different light.

Unquestionably, the inclusion of imaging factors (such as electrophysiological examination, MRI examination, etc.) or pharmacological factors in the analysis would create a more detailed picture of predictors of clinical improvement in SCI, enabling multivariate analysis. However, the retrospective character of this study did not provide an opportunity to analyze all the factors that might have an impact on post-SCI improvement.

It should also be noted that motor function is not only important for the good functioning of patients after a SCI, but also the condition of the genitourinary system, intestinal function and other SCI-related consequences. Therefore, a broader view of predicting SCI outcomes, comprising the prediction of lower urinary tract, sexual and bowel function, and autonomic dysreflexia or other complications after SCI [35,36,37] would give a more complete picture of the prognostic factors of clinical and functional improvements in SCI patients.

## 6. Conclusions

Patients with SCIs have a chance for functional improvement during ENR. Higher initial MS may increase the chances of a greater and faster functional improvement during ENR. The evaluation of initial MS in patients starting ENR after a SCI may be a good prognostic factor for functional improvement. The unquestionable advantage of the AIS scale in SCI patients is the simplicity of its execution, requiring only the skill of the examiner. Therefore, the AIS is applicable in any rehabilitation center.

Initial BI may be important in predicting clinical improvement in early SCI. However, this scale is not specific to SCI and is rarely performed in rehabilitation units for SCI patients.

The functional parameters assessed in SCI patients are among numerous possible predictors of clinical improvement. An accurate assessment of predictors of improvement in SCI patients would require a detailed analysis of other parameters. The design of a multicenter prospective study taking account of multifactorial aspects of clinical improvement after SCI could put the results of our study in a different light.

## Figures and Tables

**Table 1:** Baseline characteristics of patients (n = 38).

	Mean ± SD or N (%)	Median (IQR)	Range
Age, years	39.5 ± 17.2	33.5 (25.0 to 48.0)	from 17.0 to 78.0
Women	4 (10.5%)		
Men	34 (89.5%)		
Time from SCI to ENR initiation, weeks *	7.2 ± 5.5	5.5 (4.0 to 8.0)	from 1.0 to 27.0
Level of injury **			
C4	12 (31.6)		
C5	7 (18.4)		
C6	14 (36.8)		
C7	4 (10.5)		
C8	1 (2.6)		
Duration of ENR, weeks	14.8 ± 3.5	16.0 (15.0 to 14.0)	from 3.0 to 26.0

Abbreviations: ENR: early neurological rehabilitation; IQR: interquartile range; n: number of respondents; SCI: spinal cord injury; SD: standard deviation; *: corresponds to the length of the acute stay; **: the site of the SCI damage, which illustrates the lowest region of the spinal cord where normal motor control and sensation exist.

**Table 2:** The initial and final functional parameters in the course of early neurological rehabilitation.

Functional Parameters	Initial			Final		
	Mean ± SD	Median (IQR)	Range	Mean ± SD	Median (IQR)	Range
MS of cervical SCI (range)	16.2 ± 9.9	17.0 (8.0 to 22.0)	0.0 to 41.0	19.5 ± 11.2	19.5 (10.0 to 26.0)	0.0 to 46.0
BI	0.2 ± 0.5	0.0 (0.0 to 0.0)	0.0 to 2.0	1.3 ± 2.0	0.0 (0.0 to 2.0)	0.0 to 7.0
SCIM	9.8 ± 7.2	8.5 (7.0 to 14.0)	0.0 to 34.0	19.4 ± 12.3	16.5 (10.0 to 28.0)	0.0 to 47.0

Abbreviations: BI: Barthel Index (range from 0 to 20); IQR: interquartile range; MS of cervical SCI: ASIA (American Spinal Cord Injury) Scale Motor Scores of cervical spinal cord injury (range from 0 to 50); SCI: spinal cord injury; SCIM: Spinal Cord Independence Measure, version III (range from 0 to 100); SD: standard deviation.

**Table 3:** Change in initial vs. final functional parameters in the course of early neurological rehabilitation.

	Mean ± SD	Median (IQR)	Range	*p*-Value
MS of cervical SCI	3.4 ± 3.6	2.0 (0.0 to 5.0)	0.0 to 15.0	<0.0001
BI	1.1 ± 1.7	0.0 (0.0 to 2.0)	0.0 to 6.0	<0.0001
SCIM	9.6 ± 10.2	7.5 (0.0 to 19.0)	0.0 to 38.0	<0.0001

Abbreviations: BI: Barthel Index (range from 0 to 20); IQR: interquartile range; MS of cervical SCI: ASIA (American Spinal Cord Injury) Scale of Cervical Motor Scores (range from 0 to 50); SCI: spinal cord injury; SCIM: Spinal Cord Independence Measure, version III (range from 0 to 100); SD: standard deviation.

**Table 4:** Correlation between initial parameters and increases in the scales of interest.

Initial Parameters		Increase in MS	Increase in BI	Increase in SCIM
Initial MS	r	+0.20	+0.65	+0.36
	p	NS	<0.0001	0.028
Initial BI	r	+0.34	+0.39	+0.02
	p	0.039	0.017	NS
Initial SCIM	r	+0.10	+0.38	−0.02
	p	NS	0.018	NS
Age, years	r	+0.05	−0.19	−0.07
	p	NS	NS	NS
Time since SCI *, weeks	r	−0.17	−0.50	−0.24
	p	NS	0.002	NS
Level of SCI **	r	+0.16	+0.46	+0.26
	p	NS	0.004	NS

Abbreviations: BI: Barthel Index; MS: ASIA (American Spinal Cord Injury) Scale Motor Scores; NS: not significant; p: statistically significant; r: Spearman’s correlation coefficient; SCI: spinal cord injury; SCIM: Spinal Cord Independence Measure, version III; *: time from SCI to early neurological rehabilitation initiation (corresponds to the length of the acute stay); **: the site of the SCI injury, which illustrates the lowest region of the spinal cord where normal motor control and sensation exist.

## Data Availability

The datasets generated during the present study are available from the corresponding author upon request.
